# Nanocarriers-Assisted Needle-Free Vaccine Delivery Through Oral and Intranasal Transmucosal Routes: A Novel Therapeutic Conduit

**DOI:** 10.3389/fphar.2021.757761

**Published:** 2022-01-11

**Authors:** Bharti Mangla, Shamama Javed, Muhammad H. Sultan, Waquar Ahsan, Geeta Aggarwal, Kanchan Kohli

**Affiliations:** ^1^ Department of Pharmaceutics, School of Pharmaceutical Sciences, Delhi Pharmaceutical Sciences and Research University (DPSRU), New Delhi, India; ^2^ Department of Pharmaceutics, College of Pharmacy, Jazan University, Jazan, Saudi Arabia; ^3^ Department of Pharmaceutical Chemistry, College of Pharmacy, Jazan University, Jazan, Saudi Arabia; ^4^ Department of Pharmaceutics, Delhi Pharmaceutical Sciences and Research University, New Delhi, India; ^5^ Director Research and Publication, Lloyd Institute of Management and Technology (Pharm.), Greater Noida, India

**Keywords:** vaccine, transmucosal, nanocarrier, non-invasive, needle-free, drug delivery

## Abstract

Drug delivery using oral route is the most popular, convenient, safest and least expensive approach. It includes oral transmucosal delivery of bioactive compounds as the mucosal cavity offers an intriguing approach for systemic drug distribution. Owing to the dense vascular architecture and high blood flow, oral mucosal layers are easily permeable and can be an ideal site for drug administration. Recently, the transmucosal route is being investigated for other therapeutic candidates such as vaccines for their efficient delivery. Vaccines have the potential to trigger immune reactions and can act as both prophylactic and therapeutic conduit to a variety of diseases. Administration of vaccines using transmucosal route offers multiple advantages, the most important one being the needle-free (non-invasive) delivery. Development of needle-free devices are the most recent and pioneering breakthrough in the delivery of drugs and vaccines, enabling patients to avoid needles, reducing anxiety, pain and fear as well as improving compliance. Oral, nasal and aerosol vaccination is a novel immunization approach that utilizes a nanocarrier to administer the vaccine. Nanocarriers improve the bioavailability and serve as adjuvants to elicit a stronger immune response, resulting in increased effectiveness of vaccination. Drugs and vaccines with lower penetration abilities can also be delivered transmucosally while maintaining their biological function. The development of micro/nanocarriers for transmucosal delivery of macromolecules, vaccines and other substances is currently drawing much attention and a number of studies were performed recently. This comprehensive review is aimed to summarize the most recent investigations on needle-free and non-invasive approaches for the delivery of vaccines using oral transmucosal route, their strengths and associated challenges. The oral transmucosal vaccine delivery by nanocarriers is the most upcoming advancement in efficient vaccine delivery and this review would help further research and trials in this field.

## Introduction

The world has a long history of global disease outbreaks such as severe acute respiratory syndrome-coronavirus (SARS-CoV) in 2003, Influenza A virus subtype H1N1 causing swine flu in 2009–2010, Middle East respiratory syndrome (MERS) in 2012, and the very recent coronavirus disease-19 (COVID-19). The COVID-19 is caused by the severe acute respiratory syndrome-coronavirus-2 (SARS-CoV-2) and has been declared as a potential threat and pandemic by the World Health Organization (WHO). Exploring treatment regimens, effective therapeutic agents and vaccines for complete eradication of the virus are one of the priorities of the scientific fraternity, including all healthcare providers (Ahsan et al., 2020). Several interesting reviews concerning the development of vaccines and promising therapeutic agents for the prevention and treatment of COVID-19 have already been published recently (Ahsan et al., 2020; Li et al., 2021). Vaccines stored at room temperature lose their potency over a period of time, and continuously refrigerating vaccines, especially at subzero temperature is difficult and expensive. A huge advantage lies in the development of a vaccine that can be stored and transported at room temperature and at the same time the ecological footprint of global immunization campaigns comes down. As we have witnessed recently with COVID-19, global immunization campaigns generate millions of used syringes and needle sticks as sharp wastes, and these hazardous and non-hazardous wastes impose great burden on the ecosystem.

Needle-free, as the name indicates, aims to deliver the dose of a vaccine dose at high velocity into the dermal and subcutaneous layers without penetration by a needle. This method can be considered the newest method for vaccine delivery and holds maximum benefits in terms of waste management as generated needle waste is reduced and the potential risk of accidental needling after injection is eliminated. Needle-free vaccines for multiple and monodoses are currently being explored by researchers across the globe (Lloyd, 2000; Yang et al., 2016). This swift shift from needle-based to needle-free immunization seems to be ideal for vaccine delivery. During the pandemic/epidemic situations or global health crises, when mass vaccination is required, needle-free vaccine drug delivery provides ease and facilitates delivery, increasing safety and patient compliance, decreasing cost of medicine, and decreasing pain often associated with injectable vaccines (Giudice and Campbell, 2006; Garg and Aggarwal, 2017).

The advantages of needle-free drug delivery systems include the elimination of broken needles, needle sticks, and needle disposal, reduction of pain and stress, consistent drug or vaccine delivery, and reduced drug or vaccine volume. The disadvantages include high startup costs, infrastructure demand, and requirements for training and maintenance (Giudice and Campbell, 2006). Currently, pharmaceutical and biomedical industries are showing an upward ongoing trend towards the needle-free systems and the market size, share, demand and trend of needle-free drug delivery devices (novel needle-free inhalers, jet injectors, and transdermal patches) is predicted to increase in the coming years (Chase et al., 2008) according to application and technology used. Regular use of needles increases the risk of infection and pain as in case of type 1 diabetic patients who need regular glucose monitoring through needles. In vaccine delivery, insulin injections for diabetics, pediatric injections, and other related conditions, needle-free devices have gained much attention by providing painless delivery and negating the risk of infections.

This review article is aimed to raise awareness of needle-free vaccine delivery systems as one of the approaches for the treatment and prevention of various diseases especially in cases, where mass vaccination is required. The needle-free vaccines can be administered using the transmucosal route and extensive efforts have been made in the last few years in developing the mucoadhesive delivery systems that could deliver the drugs and antigens in appropriate concentrations. In this article, we emphasized on the oral transmucosal and intranasal route of drug/vaccine administration and various advancements made in the field are reviewed. Transmucosal delivery using nanocarriers and the approaches for the delivery of COVID-19 vaccines are also covered.

## Need for Mucoadhesive Systems

The mucoadhesive drug/vaccine delivery systems are needed owing to various advantages associated with them. Some of the benefits of using transmucosal systems include increased drug absorption due to abundant blood supply as well as good blood flow rate in the mucosal sites resulting in increased therapeutic efficacy. Other advantages include prevention of first pass metabolism resulting in increased drug bioavailability, prevention of drug degradation due to acidic environment of the gastrointestinal tract (GIT), ease of drug/vaccine administration, targeted and localized administration of the dosage form at a specific site, and provision of intimate contact between dosage form and the absorptive mucosa resulting in high drug flux at the absorbing tissue (Shaikh et al., 2011). Mucoadhesive drug delivery systems interact with the mucus layer that covers the mucosal epithelial surface as well as mucin molecules and thereby, prolong the time for which the dosage form remains at the absorption site. This phenomenon has the potential to improve controlled drug delivery in both localised and systemic drug administration by keeping the formulation in close contact with the tissues or cells at the absorption site. Furthermore, mucoadhesion has fascinated interest for the administration of various unstable bioactive molecules such as high molecular weight molecules (proteins and oligonucleotides) via parenteral routes of administration, such as ocular, nasal, vaginal, and buccal, which are difficult to administer through the oral route.

## Mechanism Underlying Mucoadhesion

**FIGURE 1:**
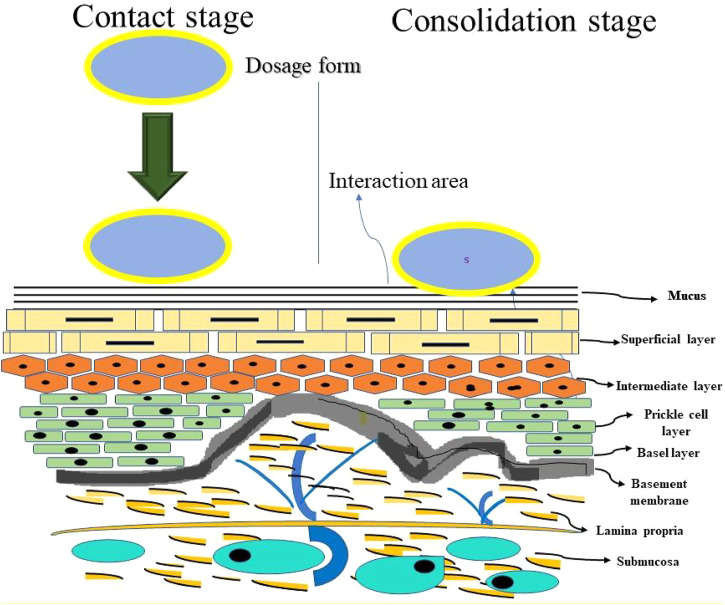
Schematic representation of the mucoadhesion mechanism.

The general mechanism of mucoadhesion includes two steps; the contact stage (between mucoadhesive polymer and the mucus membrane) and the consolidation stage (the mucoadhesive polymer is activated by the moisture present on site of administration which plasticizes the system, slowing the mucoadhesive polymer to break and link via weak van der waals and hydrogen bonds) as shown in Figure 1. Since mucoadhesion is a complex process, many theories have been proposed to explain the mechanisms of mucoadhesion involved in consolidation stage (Shinkar et al., 2012). The electronic theory states that the attractive electrostatic forces develop between the mucin and bioadhesive polymers. The wetting theory states that the bioadhesive polymer spreads on the mucous membrane and develops intimate contact. The adsorption theory states that chemical bonding is developed between mucous membrane and polymers by surface forces. The mechanical theory states that the interlocking of liquid adhesive polymer into the rough surfaces leads to adhesion. The diffusion theory states that there is physical entanglement between polymeric chains and mucin strands and the fraction theory states that it is the force required to separate two surfaces after bioadhesion is established (Boddupalli et al., 2010).

## Transmucosal Routes of Vaccine Delivery

**FIGURE 2:**
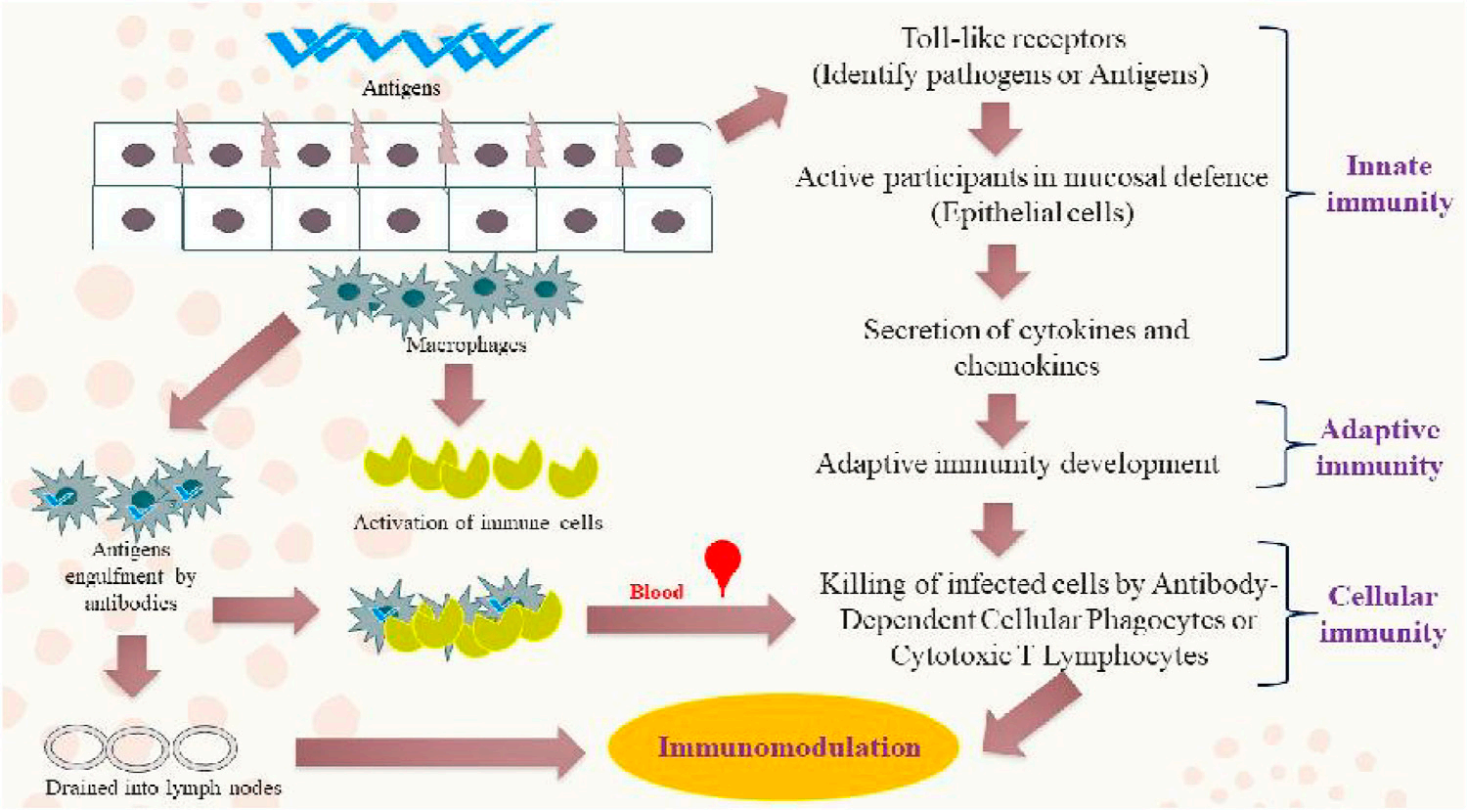
An illustration of various mucosal immune-defense mechanisms.

Apart from painless drug administration, the transmucosal route offers many advantages and showed potential and flexibility in various clinical studies (Abhang et al., 2013). Owing to its advantages over injectables and enteral methods, it can be considered an alternative to systemic drug delivery. Enhanced bioavailability projected by transmucosal route is due to the direct contact between drugs and mucosal membranes (Madhav et al., 2009). Given these advantages, vaccine injections can be replaced with needle-free oral nanovaccine delivery systems for the improvement of patient compliance. Vaccines can be administered into a patient without using a conventional needle, thereby reducing patients’ concerns and fear about the use of needles. It would also help in reducing the risk of blood-borne infections, such as human immunodeficiency virus (HIV), and ultimately increasing the patient compliance (Figure 2) (Salatin et al., 2016). In terms of prophylactic and therapeutic capacities, vaccines can induce mucosal immune responses against various diseases even cancer. Therefore, this innovative route of immunization will open a new therapeutic paradigm in the field of vaccinology and provide protection against mucosal pathogens through the transmucosal route. Since, normal microbiota is already present in the human gut, rigorous purification of bacterial by-products is not required for oral vaccines; in contrast to the vaccines delivered parenterally, where any unacceptable level of endotoxin should be monitored and taken into consideration. Therefore, combining all the advantages reported above, mucosal vaccines are aimed at facilitating the drug administration at the time of mass vaccination (Neutra and Kozlowski, 2006).

Despite all these important aspects about the oral route of drug administration, oral vaccine delivery is still not a very successful approach for presenting antigens to the body, and the reason lies in the human body physiology. The inactivating enzymes and acidic environment of the gastrointestinal tract will quickly degrade antigens before they are presented to the M-cells of Peyer’s patches (Miquel-Clopés et al., 2019). Therefore, vaccine delivery through oral transmucosal route is needed for controlled and targeted vaccine or drug delivery. In the transmucosal route, the advantages associated with local drug delivery are achieved and vaccines or drugs avoid the hepatic first-pass metabolism. Therefore, these vaccines or drugs show a decreased degree of degradation, have prolonged effects, and have a high drug flux rates in absorbing tissues (Torres-Lugo and Peppas, 2000). Mucosal vaccine delivery also induces cellular and humoral responses against mucosal infections. It is one of the most common ways of inducing desired immunity against various types of antigens and microbes in external mucosal surfaces (Kaur et al., 2018). The two types of immune responses, innate and adaptive, protect the mucosal surfaces of body interiors against infections. Innate responses prevent initial infections, whereas adaptive immune responses prevent infection from previously encountered pathogens. This local immune system contributes to nearly 80% of all immunocytes in a healthy human adult, which accumulate inside or remain in transit between the mucosa-associated lymphoid tissues (MALT). These immunocytes constitute the largest mammalian lymphoid system (Torres-Lugo and Peppas, 2000; Kaur et al., 2018) Vaccines are thought to be effective if they can successfully stimulate the innate immune system and generate a strong adaptive immunity (Kammona and Kiparissides, 2012). In vaccine delivery through the transmucosal route, application of nanoparticles or nanocarriers offers several advantages owing to their small size. Nanoparticles can infiltrate the mucosal membrane through the paracellular pathway and are limited in quantity owing to the presence of tight junctions of order 3.9–8.4 Å.

The mucosa is supplied naturally by extensive vascular and lymphatic drainage resulting in efficient absorption of vaccines/drugs locally at the site of administration. This helps bypass the hepatic first-pass metabolism as well as the presystemic elimination in the GIT. Thus, this mode of vaccine delivery is preferred by the patients and embarking this approach is a well suited one for a retentive device (Kushwaha et al., 2011). Currently, a number of mucosal vaccines have already been approved for use in humans which include the vaccines against *Poliovirus*, *Vibrio cholerae*, *Salmonella typhii* and *Rotavirus* as well as the nasal vaccine against the Influenza virus (Laffleur and Bernkop-Schnürch, 2013; Lavelle and ward, 2021). Research and advances in vaccinology have created an array of novel vaccine candidates and intriguing adjuncts that can be administered through the mucosal surface (Levine and Dougan, 1998; Kharenko et al., 2009). Oral transmucosal drug delivery systems have broad applications and are suitable for drugs that have poor bioavailability upon oral administration and are rapidly degraded by gut enzymes. Oral transmucosal medication delivery using vaccinology systems provide a number of advantages, including high patient compliance, cheap cost, convenience of administration, and the ability to prevent first-pass metabolism. Furthermore, oral transmucosal administration systems are easy to discontinue, having quick absorption, and stimulating immune responses both systemically and locally. Apart from the benefits, this delivery technique has only one disadvantage that the antigens degrades in gastrointestinal tract (GIT).

### Oral Transmucosal Route

The oral route is one of the most convenient routes of drug administration and it has now emerged as a popular transmucosal route for systemic drug delivery. This route is suitable for both rapid and sustained drug release formulations that can overcome the low permeability issues through mucosal layers (Madhav et al., 2012; Senel et al., 2012). It has become a favorable site for therapeutic application in the treatment of diseases of the oral cavity (Verma et al., 2014). Oral transmucosal drug delivery is a systemic drug delivery approach that has various advantages over parenteral and oral delivery system (Umashankar, 2015). A stratified squamous epithelium forms the outermost layer of the oral mucosa, which is followed by a basement membrane, a laminar propria, and lastly a submucosa as the innermost layer. Approximate thickness of the buccal mucosal epithelium is 40–50 cell layers, whereas the sublingual epithelium has comparatively fewer cell layers (Sangeetha et al., 1998). Delivery through oral mucosa helps evade first-pass metabolism, augments the drug bioavailability and acts as a means of rapid drug transport to the systemic circulation. Additionally, a more convenient delivery route is offered as compared to the intravenous route; hence, it is the simplest and one of the most attractive routes for vaccination (Ramirez et al., 2017).

The orally administered vaccines also generate immunological responses in parts of GIT such as small intestine, ascending colon and even in the distal tissues including salivary and mammary glands. The entry of pathogenic organisms into mucosal surfaces can be prevented by mucosal immunity rather than by systemic immunity. The majority of gut-associated lymphoid tissues (GALTs) are made up of Peyer’s patches, which serve as important inductive sites for both humoral and cell-mediated immunity. Secretory immunoglobulin A (sIgA) antibodies are produced by GALT (Hobson et al., 2003). The mucosal administration of antigens may result in the concomitant expression of sIgA antibody responses in various tissues and secretions and in the suppression of immune responses under certain conditions (Czerkinsky et al., 1999). Particulate antigens show a higher degree of response than soluble antigens because of uptake by Peyer’s patches. Therefore, a better approach is to encapsulate antigens in suitable carrier systems which would increase the uptake and prevent drug degradation in the stomach due to strong acidic environment and high enzymatic activity (Jain et al., 2011).

### Intranasal Route

Nasal nanovaccines administered through the intranasal route are optimal for mass vaccination in pandemics and are convenient for the stimulation of mucosal and systemic immune responses (Bernocchi et al., 2017). Nasal delivery improves transport across the nasal membrane and facilitates the delivery of small polar molecules, peptides, and proteins used in vaccines, such as DNA vaccines (Illum, 2003). Over the past decades, research on intranasal drug delivery route has attracted considerable interest and is recognized as a promising alternative route of drug administration. Nanocarriers can enhance the delivery of peptides, proteins, drugs, and vaccines through this route. Hydrogel polymer-based nanoparticles and nanocomposites for vaccine delivery through intranasal administration are of considerable importance to vaccinology (Gizurarson, 2012; Han, et al., 2018).

The problems associated with oral, parenteral and rectal routes of drug administrations prompted exploration of the intranasal delivery of various drugs. The incorporation of mucoadhesive polymers and absorption promoters facilitate the transport of colloidal formulations and enhance their transport across nasal mucosal barriers. Various vaccines can be delivered through the intranasal route effectively, particularly vaccines for the brain (Ali et al., 2010; Ramvikas et al., 2017). Other advantages of nasal vaccine delivery include the cost-effectiveness and patient friendly administration. The nasal cavity is extremely vascularized and has a large absorption surface area because of the abundant microvilli wrapping the nasal epithelium. Vaccination through the nasal route stimulates mucosal and systemic immune responses and is feasible for mass vaccination. It is a needle–free, syringe–free, cost-effective approach for vaccination which reduces the chances of opportunistic infections. These advantages indicate the effectiveness of intranasal route of administration in vaccine delivery for infectious diseases and certain cancers (Pabst, 2015; Kumar et al., 2016; Lobaina Mato, 2019). Intranasal vaccination using dry powder vaccine formulation has emerged as a convenient and non-invasive way to protect mucosal surfaces and to improve the storage stability (Thakkar et al., 2018).

## Methods of Characterization and Evaluation of Transmucosal Systems

Transmucosal vaccines are characterized and evaluated using a variety of *in vitro* and *in vivo* approaches and a few of them are summarized in Table 1. These strategies are utilized depending on the necessity, such as the type of formulation, type of nanocarrier, or the type of vaccines. Rats, rabbits, mice, pigs, monkeys, and other animal models have been employed in the *in vivo* experiment (Ozbilgin et al., 2014). Western blotting, gamma scintigraphy, electron paramagnetic resonance (EPR) oximetry, and enzyme-linked immunosorbent assay (ELISA) are examples of *in vivo* studies. Western blotting is used to test the integrity and specificity of proteins. Long-term stability experiments are conducted to determine the shelf life of the mucoadhesive systems. To determine the specific antibody, an ELISA test was utilised, whereas the *in vitro* methods are carried out to evaluate the basic parameters of the mucoadhesive system and to quantify the antigens using flow cytometry, confocal laser scanning microscope (CLSM) etc. (Reitan and Secombes, 1997).

**TABLE 1:** Various *in vitro/ex vivo* and *in vivo* methods for the evaluation of transmucosal vaccines.

Type of method/technique	Name of the method/technique
*In vitro/ex vivo* methods	Mucous retainability study
	Colloidal gold staining method
	Flow cytometry
	Zeta potential
	Scanning electron microscopy (SEM)
	Transmission electron microscopy (TEM)
	Particle size analysis
	Confocal laser scanning microscopy (CLSM)
	Scanning electron microscopy (SEM)
	Transmission electron microscopy (TEM)
	Electrical conductance
	Swelling properties
	Viscosity
	Refractive index
	Shear stress/Tensile strength measurement
	Fluorescent probe method
*In vivo* methods	Electron paramagnetic resonance (EPR) oximetry
	Enzyme-linked immunosorbent assay (ELISA) specific antibody measurement
	Gamma-scintigraphy
	Pharmaco-scintigraphy
	Electron paramagnetic resonance
	Use of radioisotopes
	Western blotting

Previously, these techniques have been utilized successfully to characterize and evaluate the conventional and novel transmucosal vaccines, for instance, radiolabelling technique was used to characterize the liposomal vaccine formulation of CAF01 and H56 antigens without affecting the physicochemical properties of the vaccine (Thakur et al., 2018). The *in vitro* evaluation was performed using flow cytometry which helped in the detection, identification and cell count measurement of antigens. In another study, liposome-based vaccine was developed using PspA antigens against the *S. pneumoniae* infection, which was characterized *in vitro* using cell culture method and *in vivo* using ELISA for the detection of PspA-specific antibodies (Tada et al., 2018). Flow cytomtery technique was also used to determine the uptake of PspA antigens inside the cell. The *in vitro* cell culture study has also been utilized to test the effectiveness of solid-lipid nanoemulsions prepared for the prevention of hepatitis B. The physicochemical characterization of the prepared formulation was examined using SEM, TEM and atomic force microscopy (AFM) techniques and X-ray examinations and immunological tests were performed as part of *in vivo* studies (Sahu et al., 2019).

A novel cubosome formulation using ovalbumin (OVA) as antigen and Quil-A as adjuvant was prepared and characterized using *in vitro* and *in vivo* techniques (Von Halling Laier et al., 2018). The prepared vaccine formulation was evaluated for their expression of humoral and cellular immune responses *in vivo*. The size, zeta potential and entrapment efficiency of the prepared cubosomes were determined using *in vitro* methods. Similarly, *in vitro* photon correlation spectroscopy (PCS), electron microscopy and AFM techniques were used to characterize chimeric nanoparticles-based vaccine prepared against the T cell leukemia virus, whereas the *in vivo* methods included determination of specific antibodies and cytokine responses (Kabiri et al., 2018).

Several chitosan-based nanoparticulate vaccines were also developed and characterized, as chitosan acts as a natural mucoadhesive polymer which is safe and compatible with a number of antigens. It was used to encapsulate SwIAV KAg antigen to be used against influenza virus. The *in vivo* evaluation was performed on nursery piglets which showed that the immunization was safe and effective. Particle size, zeta potential and cellular uptake experiments using *in vitro* techniques were performed on the APCs (Dhakal et al., 2018). The reverse transcription-polymerase chain reaction (RT-PCR) technique was employed to determine the RNA concentration in the vaccine. Another chitosan-based self-adjuvanting vaccine delivery system was developed recently using the anionic polymer with antigens and assessed using *in vitro* and *in vivo* techniques (Nevagi et al., 2018). This vaccine showed good efficacy against group A streptococci and were characterized *in vitro* using dendritic cell and macrophage maturation studies.

## Advantages, Limitations and Challenges of Transmucosal Vaccines

Vaccines delivered through oral and intranasal transmucosal routes have attracted much attention nowadays owing to various advantages associated with the transmucosal route as the mucosa is rich in blood supply and is relatively permeable. Dosage forms, which get adhered to the mucosa can release the vaccines in a controlled manner over a long period of time. This approach also avoids the enzymatic degradation of vaccines in the GIT and avoids the first pass metabolism in liver. However, there are several limitations or disadvantages associated with the transmucosal route which include possibility of ulcerative effects locally due to prolonged contact of the dosage form containing ulcerogenic substances. Another major limitation with the development of oral transmucosal systems is the lack of good model for *in vitro* screening in order to identify the drugs suitable for administration through this route. Additionally, the taste and irritancy of the dosage form might have an effect on the patient acceptability and compliance. Eating and drinking is also prohibited in case of vaccines delivered through oral transmucosal route which further affects the patients’ compliance. Various advantages, limitations and challenges associated with the development of oral and intranasal transmucosal vaccines are summarized in Table 2.

**TABLE 2:** Advantages, limitations and challenges associated with various transmucosal route vaccine delivery systems.

Types of transmucosal delivery system	Advantages	Limitations	Challenges	References
Oral transmucosal vaccine delivery systems	Easy and self-administration Several dosage forms options Bypass first pass metabolism Fast onset of action from mucosal site Provides protection to the vaccine from acidic environment in gut and digestive enzymes High target specificity Controlled release of vaccine Rapid absorption because of increase blood supply Increased patient compliance Excellent accessibility	Small surface area for absorption Dissolution problem in patients with dry mouth Unpalatable May not be suitable for vomiting and unconsciousness patients Rapid clearance of administered vaccine by saliva Vaccines should pass from the mucosal barrier to reach site of action Inconvenient for some patients Irritation of oral mucosa Small dose limit	Protecting biological drugs such as peptides and proteins from enzymatic degradation Developing drugs or delivery systems that overcome the permeability barrier Polymeric vaccine delivery by parentral route is invasive and painful Limited efficient drug delivery systems	Sankar et al. (2011) and Cho et al. (2021)
Intranasal vaccine delivery systems	Highly vascularised mucosa Easy accessibility Needle free vaccination site Faster onset of immune response Induction of cross-reactive antibodies Reduction in the number of vaccinations required Lower cost Nanopowder has increased stability and ability to target further into the nasal cavity	Variability in dosing Nasal obstruction/inflammation Special applicators are sometimes required for nanopowders limiting the ease and increasing the cost of administration	High mucociliary clearance Narrow nasal passage Complex nasal geometry Negatively charged nanoparticles not compatible with mucosal delivery	Yusuf and Kett (2017)

## Vaccine Delivery Systems

### Conventional Vaccine Delivery Systems

Conventional vaccines are inadequately strong against severe and sometimes life-threatening medical conditions as the incorporation of live or killed microbes fail to produce desired immune responses. Research on nanovaccinology has rapidly advanced because of the attractive properties of nanoparticles such as size, shape, charge, biocompatibility, biodegradability, high specificity, capability to modulate vaccine composition, overcome natural barriers and control the antigen release (Yadav et al., 2018). Fast-dissolving buccal films can be ideal drug delivery systems for vaccines owing to various advantages such as capability to evade the first-pass effect, easy administration, cost effectiveness, and easy preparation steps (Uddin et al., 2019).

**FIGURE 3:**
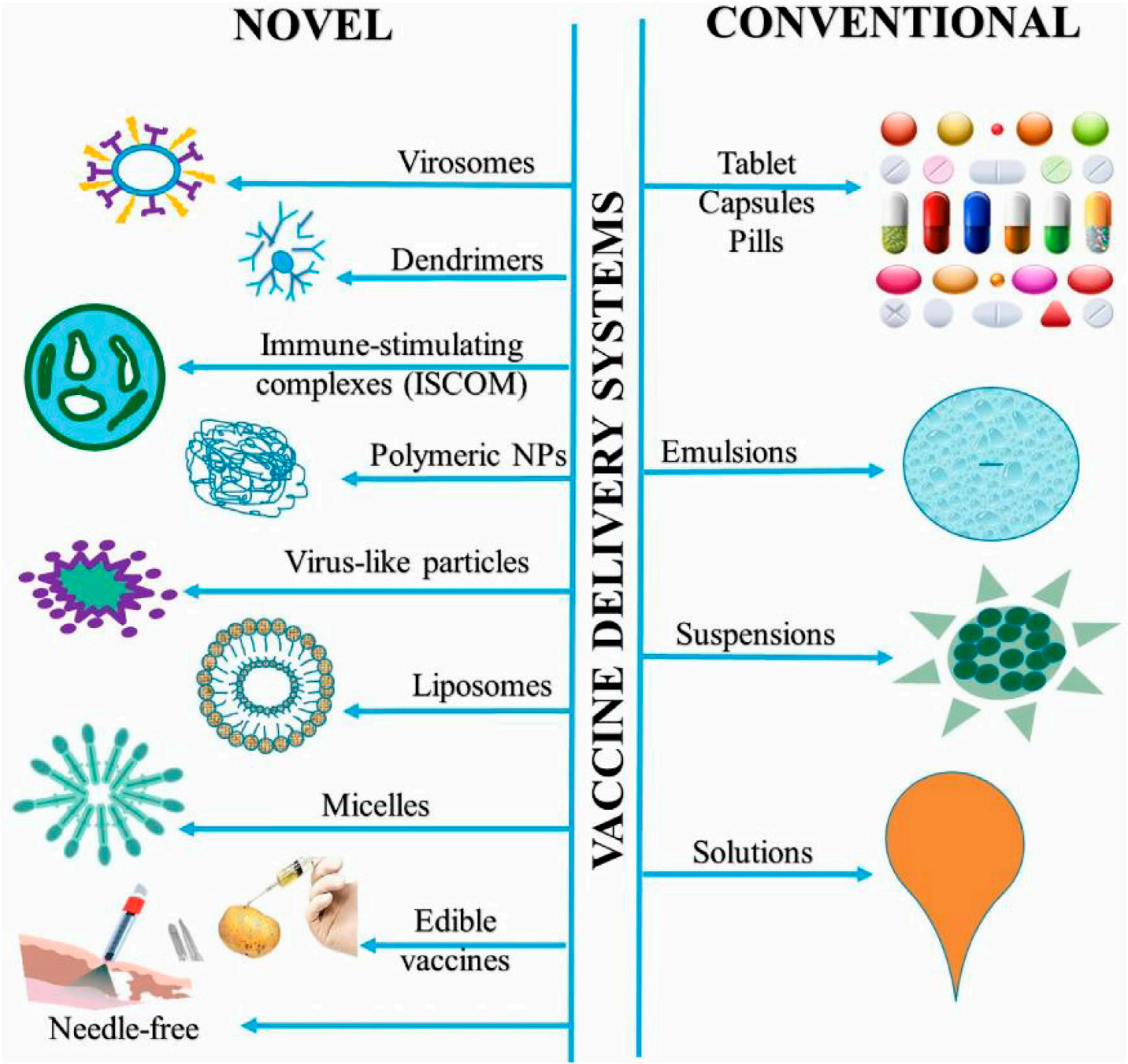
Diverse novel and conventional vaccine delivery systems.

Vaccines can be delivered through both conventional and novel delivery systems and efforts are being made for developing needle-free vaccine formulations against all forms of diseases. Recently, a novel method for stabilizing live viruses and other biological medicines in a rapidly dissolving film delivery system was developed that does not require refrigeration (Bajrovic et al., 2020). The goal was to develop a needle-free method for vaccine delivery while maintaining the shelf-life stable. The film could be administered through the mouth and was inexpensive as well as stable. This idea can make vaccine campaigns affordable as large quantities can be shipped and distributed easily. This new technology can improve global access to vaccines by making manufacturing and distribution easy. Edible vaccines are an innovative approach of administering immunizations by oral route and it has gained considerable interest recently because of their multiple benefits including patient friendliness and compliance among the top ones (Lakshmi et al., 2011). Figure 3 shows various conventional and novel vaccine delivery systems and each method has its own benefits and limitations. Needle-free delivery and mucosal delivery of vaccines are the latest and newest ways of delivery.

### Nanocarriers for Transmucosal Delivery of Vaccines

Major developments in novel vaccines with low cell toxicity and high specificity are warranted owing to the rapid emergence of infectious and chronic diseases, the latest of which is COVID-19. Low immunogenicity and complex processing steps are often the limiting factors in their development. The advantages of nanovaccines, including polymeric nanoparticles, liposomes, micelles, and nanofibers, are reduced administration dose, improved delivery efficiency, targetability, precise stimulation of immune responses, and desired biocompatibility (Yang et al., 2016; Sharma et al., 2017). A novel non-invasive vaccine was developed through the immobilization of the Pertusis toxin in electrospun polymer (polyvinylpyrrolidone) nanofibers for effective long-term immunity against whooping cough through dermal pathway, thus overtaking oral and injectable vaccines (Gawade et al., 2008). Chitosan is an attractive polymer that can be used in the development of nanovaccines meant to be administered through mucosal routes for massive and painless immunization (Gourapura and Renu, 2020). Electrospun nanofibers made of mucoadhesive chitosan and polyethylene oxide have been evaluated for sublingual drug delivery. The mechanism behind the strong mucoadhesion of these nanofibers were the intermolecular interactions present between the polymer chitosan and mucin of bovine submaxillary glands leading to the adhesion (mucoadhesion) of these nanofibers to the porcine sublingual mucosa *ex vivo* (Stie et al., 2020). Vaccine delivery through novel drug delivery carriers, such as discosomes, dendrimers, niosomes, hydrogels, liposomes, nanofibers, and nanowafers, through non-invasive delivery routes, such as ocular, nasal, oral, pulmonary, transdermal, and rectal, has become an important research topic (Kim et al., 2012).

Vaccines require high levels of safety, cost-effectiveness and stability, which can be achieved through the use of delivery cargos. Nanocarriers have attracted considerable interest as optimal carriers for the transport of bioactive compounds to mucosal sites because of their unique properties, such as size and good interaction and penetration effects. Nanotechnology has created its space in every field of science including vaccinology (Herbst-Kralovetz, 2014). Nanocarriers designed with vaccines or antigens are projected to be more immunologically effective than the conventional dosage forms because they may be tailored to target specific areas and remain at the desired site of action for extended periods of time. In addition to the systemic immune response, nanocarriers of antigens and vaccines can activate immunization across several mucosal barriers via mucosal route, which is the body’s essential first line of defense (Shahiwala et al., 2007). Although, various types of nanocarrier-based formulations have been assessed for the mucosal administration of non-living vaccines, most of them are still in the preclinical development phase and none has entered clinical trials. Therefore, the design and development of novel, effective, mucosal, prophylactic and therapeutic vaccines against mucosal pathogens (e.g., HIV, tuberculosis, herpes viruses, and influenza) and pathogenic enteric and fungal contagions allowing sufficient uptake of adrenocarcinoma gastric cell lines (AGS), are needed. These vaccines can stimulate innate immune cells and elicit mucosal and systemic adaptive immunity (Kammona et al., 2017).

A number of nanoparticulate carriers are well established for conventional drug delivery, but the commercial application of mucosal vaccine delivery is still encountering many challenges. For successful translation to clinic, nanocarriers should be capable of protecting antigens from the harsh gastric environment, tailorable to meet targeting demands, and capable to accomplish the desired release profile of antigens. These barriers have been addressed to some extent by nanocarrier-based antigen delivery; however, many issues regarding dosing for targeted mucosal vaccine remain unresolved. Nanocarriers serve as a potential field of research for the development of promising future mucosal vaccines (Kim et al., 2014). Transmucosal drug delivery can be improved by using chemicals which can reduce the intrinsic resistance shown by the physiological barriers. These chemicals can be the enzyme inhibitors or the permeation enhancers; however, they might bring the risk of infections as they can reduce the mucosal protective capacity (Csaba et al., 2006).

Mucosal nanovaccines are highly desirable as they are administered through the non-parenteral route, are of considerably reduced cost, and show increased patient compliance (Rosales-Mendoza and González-Ortega, 2019b). Gold-based vaccine candidates against influenza and tetanus are showing promising results at preclinical levels in animal models (Rosales-Mendoza and González-Ortega, 2019a; Dykman, 2020). Nanoparticle carriers as nanovaccines have emerged as promising tools for the intraepithelial delivery of biomacromolecules through mucosal surfaces (De la Fuente et al., 2008). Vaccination is of great significance to the improvement of human life expectancy and control of fatal diseases. Advancements in vaccinology are needed in addressing the emergence of new infectious diseases such as COVID 19. Great potential is shown by the self-assembled nanoparticles in delivering the antigens effectively by functioning as antigen vehicles as intrinsic adjuvants. They can also enhance the antigen presentation, thereby increasing the humoral and cellular immune responses (Pan and Cui, 2020). Conventional vaccines are easily degraded and sometimes get cleared from the site before the desired action. Vaccine components are encapsulated by the polymer-based nanocarriers which leads to the protection of sensitive components, incorporation of mucosal adjuvants, augmentation of immunological responses, targeting the mucosal immune system and improvement in the efficiency of mucosal vaccines (Sharma et al., 2015).

Recent developments in nanotechnology systems have sparked significant interest in the field of vaccine development. Antigens for vaccines can either be encapsulated or surface-adsorbed on the nanocarrier surface, depending on the application. It is more efficient to deliver vaccines using nanocarriers as it produces greater immune response in comparison to the conventional vaccines (Reichmuth et al., 2016). Furthermore, when vaccines are delivered using nanocarrier, they are directly targeted to tissue and their oral bioavailability is improved. A wide range of nanoparticles have been investigated in the field of vaccinology that are delivered through the mucosal surface (Thakur and Foged, 2020). It includes lipid-based nanoparticles, natural or synthetic polymer-based nanoparticles, microbubbles, nanotubes, chimeric peptides and many more.

When employed for vaccine delivery, nanocarriers must meet three primary requirements: they must protect antigens, carry the antigen through mucosal cell, and deliver the antigen to the anti-protecting cell. There are numerous aspects which can have impact on the effectiveness of nanocarriers, either directly or indirectly. Particle size, surface charge, stability or solubility in a biological environment, polymers crystallinity, ratio of co-polymers, bio-adhesiveness, nature of additives and glass-transition temperature are some of the factors to consider during development of nanocarrier-based vaccine delivery systems (Kamaly et al., 2016). Many of these characteristics have an impact on the rate at which antigen is released from nanocarriers and the rate at which it is taken up by macrophages. Studies concerning vaccine delivery using multiple nanocarriers through transmucosal routes such as oral, nasal, and pulmonary are summarized in Table 3.

**TABLE 3:** Nanocarriers utilized to deliver antigens through the oral and intranasal transmucosal routes.

Nanocarrier	Vaccine/antigen	Route	Observation	References
Chitosan nanoparticles	SARS-CoV-2	Intranasal	Intranasal delivery of Receptor bindind domain of - *N*,*N*,*N*-trimethyl chitosan nanoparticles into mice induced robust local mucosal immunity, as evidenced by the presence of IgG and IgA responses in BALs and the lungs of immunized mice	Jearanaiwitayakul et al. (2021)
Graphene oxide nanoparticles	Influenza	Intranasal	Immunization with GP oxide nanoparticles, conferring protection against homologous and heterologous viruses	Dong et al. (2021)
Chitosan nanoparticles	*Salmonella enteritidis*	Oral	Enhanced mucosal IgA antibody, cellular immune response, TLRs gene expression	Renu et al. (2020)
Mannose conjugated chitosan nanoparticles	*Salmonella enteritidis*	Oral	Enhanced cell mediated immune response, TLRs and balanced Th1 and Th2 cytokine gene expression; reduced Salmonella challenge load in the cecum	Han et al. (2020)
Chitosan conjugated nanoparticles	*Salmonella enteritidis*	Oral	Induced cross-reactive IgG and mucosal IgA antibodies, cytokine gene expression; lower heterologous challenge bacterial load in liver and spleen	Acevedo-Vilanueva et al. (2020)
Solid lipid nanoemulsions	Hepatitis B	Oral	Lyophilized nanoparticles were used and found to be a novel strategy for immunological protection against hepatitis B	Sahu et al. (2019)
Dendrimers	HIV-1	Intranasal	The IgG and IgA responses in serum as well as nasal washes were shown to be improved	Rodríguez-Fonseca et al. (2019)
Liposome-based cationic adjuvant	Synthetic mycobacterial cordfactor (H56/CAF01)	Pulmonary	This vaccine was found to be effective against Tuberculosis and evenly distributed to the lungs	Thakur et al. (2018)
Liposomes	Pneumococcal vaccine	Intranasal	Highly effective mucosal vaccine system for the delivery of pneumococcal vaccine	Tada et al. (2018)
Cubosomes	Ovalbumin	Oral	Spray drying technique was reported to be a viable approach to make dry powder nanoparticulate vaccine formulations	Von Halling Laier et al. (2018)
Chimeric peptide	Human T-lymphotropic virus-1 (HTLV-1)	Intranasal	Correct conception, manufacture, and immunization of multi-epitope vaccine were required for the development of an effective HTLV-1 vaccine	Kabiri et al. (2018)
Lipid-polymer hybrid nanoparticles	Chlamydia vaccine (CTH522)	Intranasal	An effective technique for modulating the strength of mucosal vaccination responses	Rose et al. (2018)
Microbubbles	Ovalbumin	Intranasal	Micobubbles were found to be effective in suppressing the allergic asthma	Corthésy and Bioley, (2017)
Virosomes	Asian avian influenza A (H5N1)	Intranasal	This vaccination had been demonstrated to be effective against influenza virus due to the development of a protective cell-mediated immune response	Ebensen et al. (2017)
Polymeric hybrid micelles	Ovalbumin	Intranasal	A potential multifunctional polymeric delivery system for nasal vaccination was developed	Li et al. (2017)
Liposomes	Leishmania amazonensis antigens (LaAg)	Intranasal	Intranasal administration was shown to be effective in increasing lymphoproliferative immune responses	Leal et al. (2015)
Nanoemulsion	Respiratory syncytial virus (RSV)	Intranasal	This vaccine was found to be both safe and efficacious for immunization in a variety of animal models	O'Konek et al. (2015)
Lipid nano capsules	Ovalbumin	Pulmonary	This vaccine had the potential to stimulate powerful T cell responses, which can aid in the protection of mucosal surfaces	Li et al. (2013)
Polyethyleneimine	HIV-1 CN54gp140	Intranasal	This vaccination had been shown to be effective against pulmonary viral infection	Mann et al. (2013)
Nanoemulsion	Influenza A vaccine	Intranasal	Developed for influenza virus vaccine, this system could be used as a non-toxic mucosal adjuvant	Myc et al. (2013)
Chitosan nanoparticles	Bovine serum albumin	Oral	The mucosal secretions had considerably greater serum IgG titres and sIgA levels	Jain et al. (2010)
Bilosomes	Influenza A antigen	Oral	This system was found to be effective against infection due to Influenza A virus	Mann et al. (2009)
PLGA nanoparticles	Hepatitis B	Oral	These nanoparticles were found to be an effective oral carrier for hepatitis B virus	Gupta et al. (2007)
Nanoemulsion	Recombinant anthrax vaccine	Intranasal	When compared to currently available vaccine, this approach was proven to be efficient against *Bacillus anthracis* spores and showed lesser side effects	Bielinska et al. (2007)
Niosomes	Tetanus Toxoid vaccine	Oral	Niosomes carrying TT vaccine were found to be effective in activating the cellular and humoral immune responses	Jain and Vyas, (2006)
Chitosan nanoparticles	Tetanus toxoid (TT) vaccine	Intranasal	This nanoparticle was found to effective for immunization using TT vaccine	Vila et al. (2004)
Cationic liposomes	Plasmid DNA	Intranasal	Cationic liposomes carrying plasmid DNA were found to be effective in successfully activating cellular, humoral and mucosal immune responses	Wang et al. (2004)
Virosomes	Influenza vaccine	Intranasal	High rate of mucosal protection and played a substantial role in reducing influenza-related morbidity and mortality	Durrer et al. (2003)

SARS-CoV-2: severe acute respiratory syndrome coronavirus -2; IgG: immunoglobulin G; IgA: immunoglobulin A; sIgA: serum immunoglobulin A; BAL: bronchoalveolar lavage; GP: graphene; TLRs: toll-like receptors; Th1: helper T cells 1; Th2: helper T cells 2; HIV: human immunodeficiency virus; PLGA: poly(lactic-co-glycolic acid).

## Needle-Free Vaccine Delivery System

The current concept of nanovaccinology revolves around needle-free and single-dose vaccination (Vicente et al., 2010). The benefits of needle-free vaccination include self-vaccination by adults at home, reduction in the risk of repeated use of injection devices, and reduction in the risk of cross contamination. These benefits also reduce cost and improve the vaccine coverage, making the vaccines affordable and safe.

**FIGURE 4:**
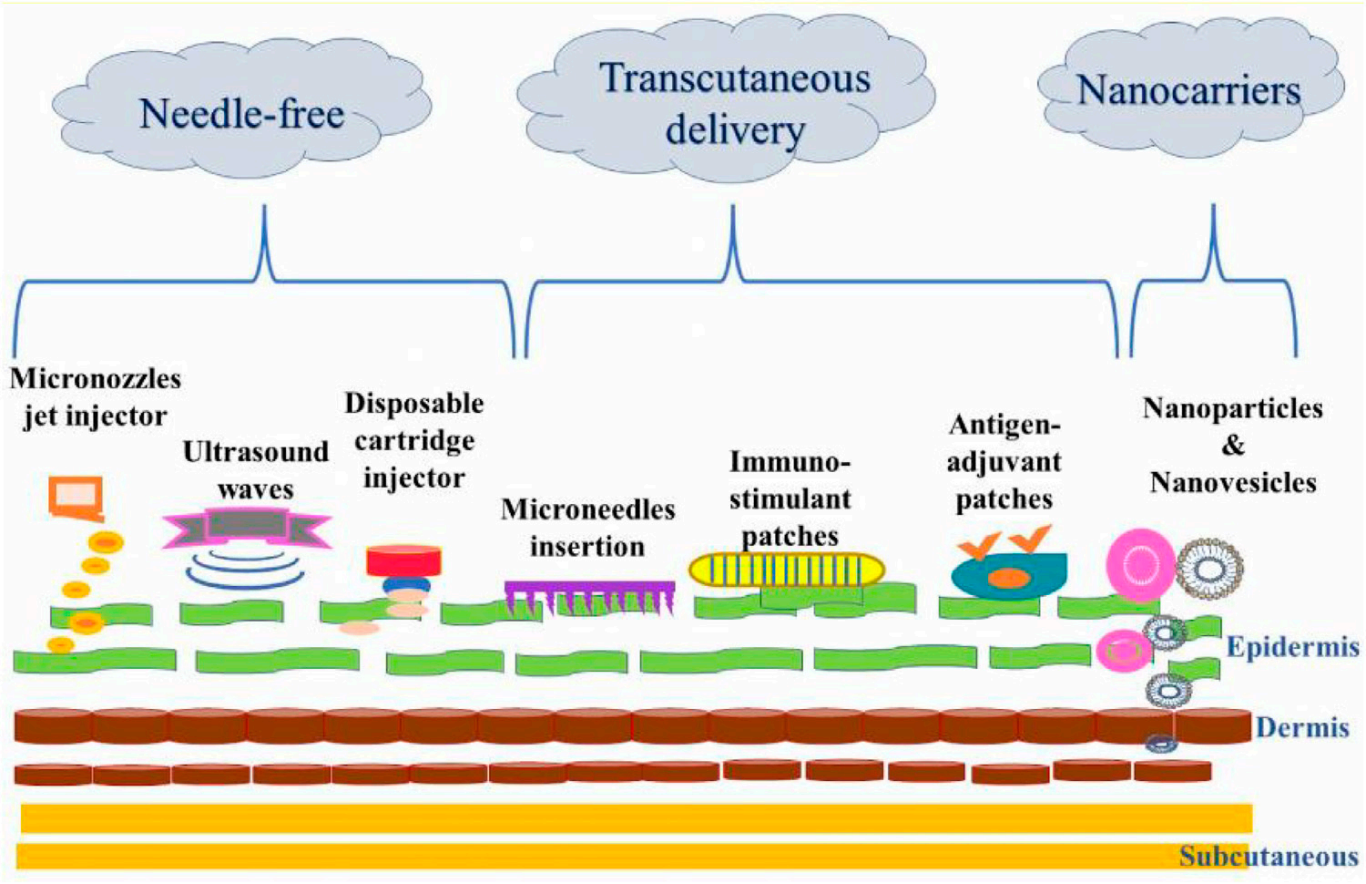
Depiction of various transcutaneous, needle-free and nanocarrier approaches for mucosal immunization.

Oral, nasal, ocular, aerosol, rectal, vaginal and nasal vaccines are delivered through various types mucosal immunization approaches as shown in Figure 4. These approaches include micronozzle jet injector, ultra sound waves, disposable cartridge injector, microneedle insertion, immunostimulant patches, antigen adjuvant patches and particulate systems. The needle free vaccine delivery system involves a micronozzle jet injector, ultra sound waves, and disposable cartridge injectors. The transcutaneous delivery system includes stimulant patches, microneedle insertion, and antigen adjuvant patches. The particulate devices is included in the nanocarrier system. The implantation of an immunostimulant adjuvant patch improves the humoral and cellular immune responses to DNA vaccination. With a frequency of 20 kHz, ultrasonic waves produce an inaudible sound and are known to improve the permeability of cells. Using nanoparticulate systems, microparticles, and lipid-based systems, antigens can be targeted to macrophages and dendritic cells with greater precision and effectiveness. Moreover, Microneedles are a painless way to distribute antigens across the skin. In the microjet injector, a piezoelectric actuator is utilised to drive a plunger in an acrylic micronozzle. The actuator is powered by electricity and aid in deliver vaccines. The term “adjuvant” refers to a substance that is added to some vaccinations to enhance the development of a stronger immune response in those who receive the injection. Improved knowledge regarding the immune system and its responses to vaccination continued to improve vaccine delivery technologies towards the needle-free approach. Few promising methods for oral vaccination include the use of attenuated bacteria as vectors and transgenic plant edible vaccines (Giudice and Campbell, 2006). Oral vaccination approach seems to be beneficial for the pharmaceutical industry, as well as the economy of developing countries as it is useful during mass immunization programs. It bypasses needle injury and prevents complications and blood-borne infections which may arise because of the multiple use of a single needle (Ren et al., 2014; Ravi et al., 2015). The successful delivery of the DNA vaccine in clinical trials is a promising method for stimulating gene expression and immune responses because of the presence of Langerhans cells (Roy et al., 2000). This new approach was aimed at lowering the economic burden and easing the logistical burden imposed by immunization programs (Ekwueme et al., 2002). It induced the robust serum antibody responses that were augmented by different immune modulators stimulating innate immune system. In mice, this method provided protection against lethal challenge with a highly pathogenic avian H5N1 influenza virus (Garg et al., 2007). Advances in the drug delivery of vaccines through the skin and various mucosal surfaces have prompted the identification of effective immunization routes which optimize protective immunity and reduce the risks and drawbacks of needle-based vaccination. Long-term therapy using small doses could be provided and the need for the administration of booster doses could be avoided using this technique making this a patient-friendly method.

### Non-Invasive Vaccine Delivery Through Mucosal Route

Vaccines can be categorized based on route of administration (oral, intranasal, intramuscular, and subcutaneous), delivery system (conventional, such as films, patches, and drops; novel, such as nanoparticles, liposomes, bilosomes, and polymeric nanogels) and antigen (dead, attenuated, inactivated pathogens, DNA based, liposome based, and virus particle based). The alternative routes of administration through the oral cavity, such as buccal or sublingual, have gained advantages in systemic and mucosal immunity. Oral mucosal vaccines are easy to prepare and safer than parenteral vaccines (Ferreira et al., 2013). Mucosal surfaces act as entry ports to infectious agents into the human body. Thus, mucosal vaccines are needed for their suppression and control and administration of vaccines through the mucosal route is a perfect strategy to evoke efficient immune responses as the parenterally administered vaccines induce poor mucosal immunity (Mody et al., 2015). Mucosal vaccines have shown promising results and elicit mucosal and systemic immune responses, providing protection against pathogens that mainly infect through mucosal routes. The limitations encountered by the mucosal vaccine delivery systems, such as pH and degradation by enzymes, can be overcome by the use of nanocarriers with the advantages of being nano-sized, biodegradable, and biocompatible and have cell-specific targeting ability. These nanocarriers enhance immunogenicity and prolong retention time at mucosal sites (Kim et al., 2013).

Extensive efforts have been made to develop effective vaccine antigen delivery systems that increase uptake by local antigen-presenting cells (APCs), resulting in protective mucosal immunological responses. Effective mucosal adjuvants and efficient delivery systems are vital for the successful mucosal vaccine delivery (Rhee, 2020). Non-invasive drug delivery is a desirable feature in healthcare systems for many reasons, including improved safety and compliance, decreased pain, easy and fast delivery, and reduced treatment cost as compared to the invasive route (Bajracharya et al., 2019). Intranasal, buccal, sublingual, pulmonary, and transdermal routes are increasingly becoming the sites of choice for the development of non-invasive systems (Bajwa et al., 2006). The traditional intramuscular, intravenous, oral and rectal drug administration routes may be unsuitable to certain patient populations. In comparison to the orally delivered medications, transmucosal methods avoid hepatic first-pass metabolism and gastrointestinal degradation. It also combats the issues that are frequently related to the orally delivered medications (Ashburn and Stanley, 1991). The human mucosa is in close proximity with blood circulation and can be explored as a possible delivery site. However, the complexity and challenges associated with it should be considered as the anatomy and physiology of each mucosal site is different from the other. Hence, considerable attention should be given in the selection of the disease and the approach for drug delivery to the target. Moreover, age and sex of a patient and kinetics and longevity of therapy must be presumed (Goyal et al., 2018). This non-invasive delivery is possible for molecules such as proteins and peptides through nasal, buccal, vaginal, transdermal, and pulmonary routes, reducing the cost of clinical use because of the possibility of self-administration of drugs or vaccines by patients (None et al., 2011; Shadab et al., 2017). One of the important considerations with non-invasive drug delivery systems is that they should be compatible with regulatory approved excipients (Anselmo et al., 2018). Still, the non-invasive delivery route pose a great challenge in protein delivery as the bioavailability of proteins administered through non-invasive delivery is lower than that in the parental route (Sachdeva et al., 2016).

## Recent Advancements in Transmucosal Vaccine Delivery

**FIGURE 5:**
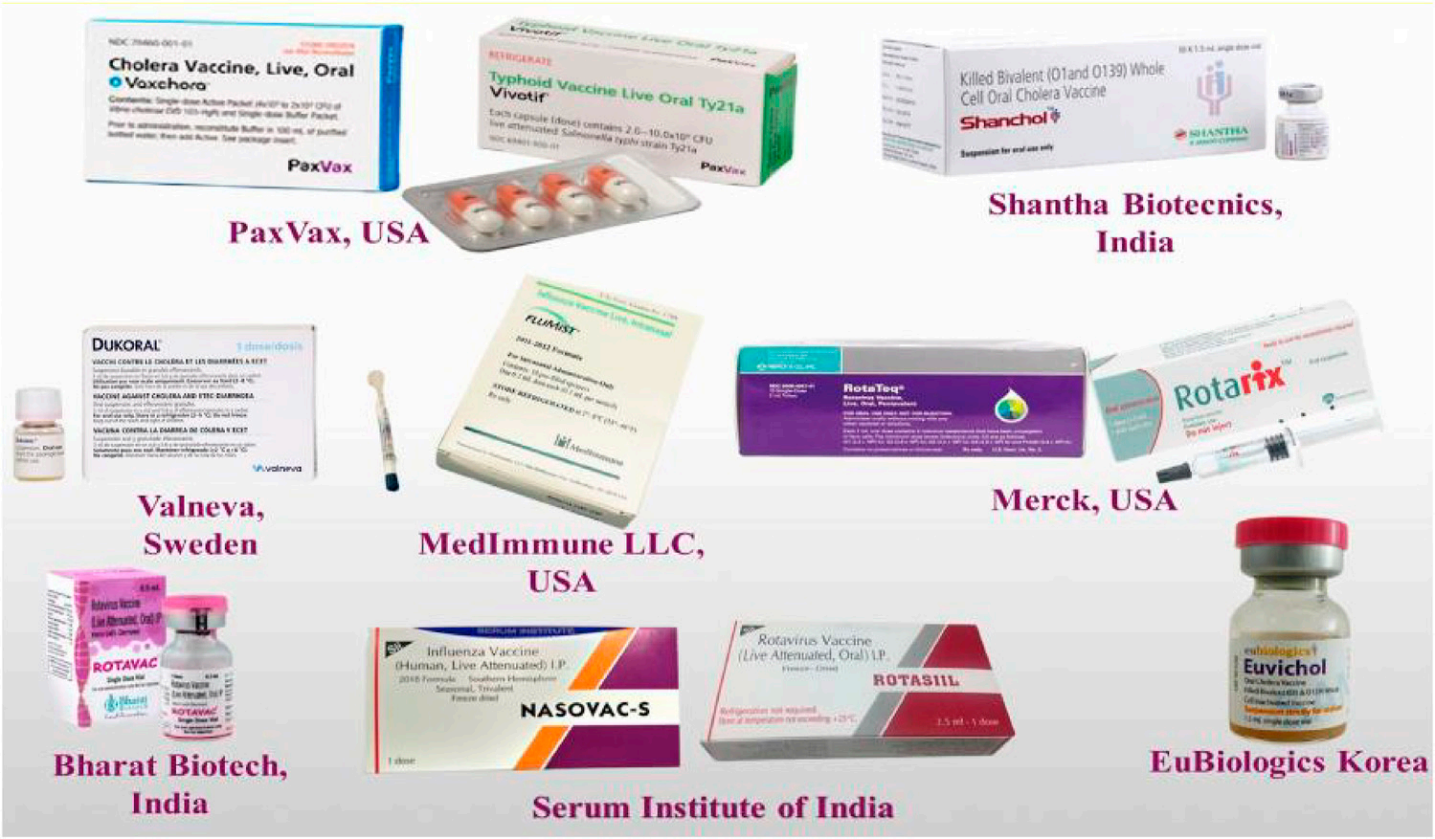
Figurative collage of various commercially available needle-free vaccines.

Although, only few transmucosal vaccines made it to the market till now, however, current technological advancements and improved understanding of the concepts of mucosal immune responses have increased the optimistic approach on the future of transmucosal vaccines. Some of the marketed oral and intranasal transmucosal vaccines along with their manufacturers are presented in Figure 5. The subcomponent vaccines which are often protein-based are comparatively safer than the live attenuated vaccines. They are more stable and are easy to manufacture; however, nontoxic and clinically safe adjuvants are needed to be added to make them more effective. Particularly, the liposome technology is considered to be ideal for combining the antigens and adjuvants to develop an effective transmucosal vaccine (Bernasconi et al., 2016). Overall, particulate delivery technologies have an irrefutable space in the development of vaccine formulations with adjuvants. Generally, the trends on this research are on the use of polysaccharides, polyesters and lipids on one hand and designing structures mimicking the pathogenic agents on the other hand (Cordeiro and Alonso, 2016). The next generation vaccines can be successfully applied if the protection can be achieved by administering minimal number of doses and a practical approach leading to the induction of mucosal immunity can be developed. Owing to these reasons, a biocompatible and biodegradable co-polymer poly (DL-lactide-co-glycolide) was used to formulate vaccine-containing microspheres.

In order to provide maximum vaccine effects at the mucosal site for a prolonged duration, it is desirable for the mucosal vaccine to remain adhered with the mucosal membrane. Thus, the vaccine delivery through the mucosal route (oral, nasal, vaginal, rectal and ocular) requires the use of mucoadhesive polymers such as Chitosan, Cellulose, Eudragit and Hybrid polymers for the production of the mucoadhesive systems. These mucoadhesive polymeric particles for mucosal vaccine delivery pose numerous advantages such as protection of antigens from degradation, controlled release of the loaded vaccine, prolonged residence time of the antigen at the target site and rapid induction of mucosal and systemic immune responses (Cho et al., 2021).

The use of adjuvants in the delivery of vaccine is critical as they boost the immune responses when used in conjunction with specific antigens and increase the effectiveness of vaccines. Additionally, adjuvants are non-specific immunopotentiators and they are able to alter the nature of immune responses. The choice of adjuvants in the development of vaccine depends on several factors including the type of disease, route of vaccination, nature of antigen and the type of immune response. The use of incorrect adjuvant might result in a reduction in the vaccine efficiency. Till date, only a few adjuvants are approved for human vaccines which include alum (Lee and Nguyen, 2015), heat-labile enterotoxin (LT) (Tregoning et al., 2018), MF59, AS03 and AF03, a squalene-based adjuvant (Wilkins et al., 2017; Ko and Kang, 2018; Wang and Xu, 2020) and AS04, a mixture of alum and lipid (Wang and Xu, 2020). These adjuvants have been shown to enhance cell-mediated as well as humoral immunity.

Recently, ovalbumin was used as an antigen in the development of microneedles where OVA-coated microneedles were prepared with and without cholera toxin (CT) as adjuvant along with disks without tips (Oh et al., 2021). These delivery systems were administered in two doses and the IgG levels were determined after 2 weeks of the second administration. Polylactic acid (PLA) was used to manufacture the structures and coating was made using carboxymethyl cellulose (CMC). The prepared microneedles were shown to successfully enter the mucosa and subsequently, the coating was dissolved to release the antigen. When the efficacy and release of microneedles and disks were compared, it was found that the microneedles were able to diffuse mucosal layer in 20 min, while disks diffused very slowly and should be in the contact with mucosa for several hours. In another study, an orodispersible film (ODF) containing H5N1 whole inactivated influenza virus vaccine was developed (Tian et al., 2020). Sugars such as trehalose and pullulan were used as stabilizing excipients which helped in maintaining the antigenicity of vaccine during preparation as well as during storage. These antigens incorporated as ODF showed excellent stability even in challenging conditions for 4 weeks.

However, the use of microneedles is associated with issues such as higher cost, slower antigen delivery and inability to generate mucosal immunity which is important especially in case of immunity against pathogens. To overcome these problems, a novel non-invasive buccal mucosa immunization strategy called “MucoJet” was developed recently, which is a three-dimensional microelectromechanical system-based vaccine/drug delivery technology (Munang’andu, 2018). MucoJet can be administered orally and is placed in the oral cavity adjacent to the buccal mucosa. It utilizes a self-contained gas-generating chemical reaction which produces a liquid jet of vaccine with high pressure. The vaccine jet ejected from the device can penetrate the buccal mucosa which was demonstrated on rabbits using OVA antigen. Anti-OVA IgG and IgA were shown to be present in rabbits treated with OVA using MucoJet technology.

The microspheres of size 1–10 microns loaded with the toxoid vaccine of *Staphylococcal* enterotoxin-B were successfully used to immunize mice subcutaneously. These microspheres showed 500-folds increase in the circulating anti-toxin response. The strength of adjuvant activity was shown to be dependent upon the size of microspheres and should not be more than 10 microns in diameter and requiring that the antigen is present within the particles (Fujkuyama et al., 2012). These microparticles prepared from the biodegradable polymer poly(DL-lactide-co-glycolides) are presently under extensive preclinical assessment as vaccine adjuvants. The controlled release features of these microparticles can further be utilized to prepare single-dose vaccines (O’Hagan, 1998). The achievement of mucosal and systemic immune responses or the induction of mucosally-induced systemic immunologic hypo-responsiveness (mucosal tolerance) is dependent upon the nature of the antigenic simulation of specific lymphoid structures and the eventual expression of Th1 versus Th2 or Th3 T-cell responses as well as the expression of pro-inflammatory versus immunoregulatory cytokines (Ogra et al., 2001). Regardless of the attractiveness of transmucosal vaccines for the development of mucosal and systemic immune responses without any inconvenience, further progress is needed in the induction of protective immunity, cost reduction, and stability (Azegami et al., 2014). Novel strategies concerning mucosal vaccines are aimed on developing non-replicating subunit vaccines, recombinant vaccines and the utilization of mucosal adjuvants (Simerska et al., 2009). Furthermore, effective targeting of particulate vaccine formulations combined with strong immunomodulation should improve the efficacy of non-living subcomponent mucosal vaccines (Lycke, 2012).

### Vaccine Delivery Approaches in COVID-19

**FIGURE 6:**
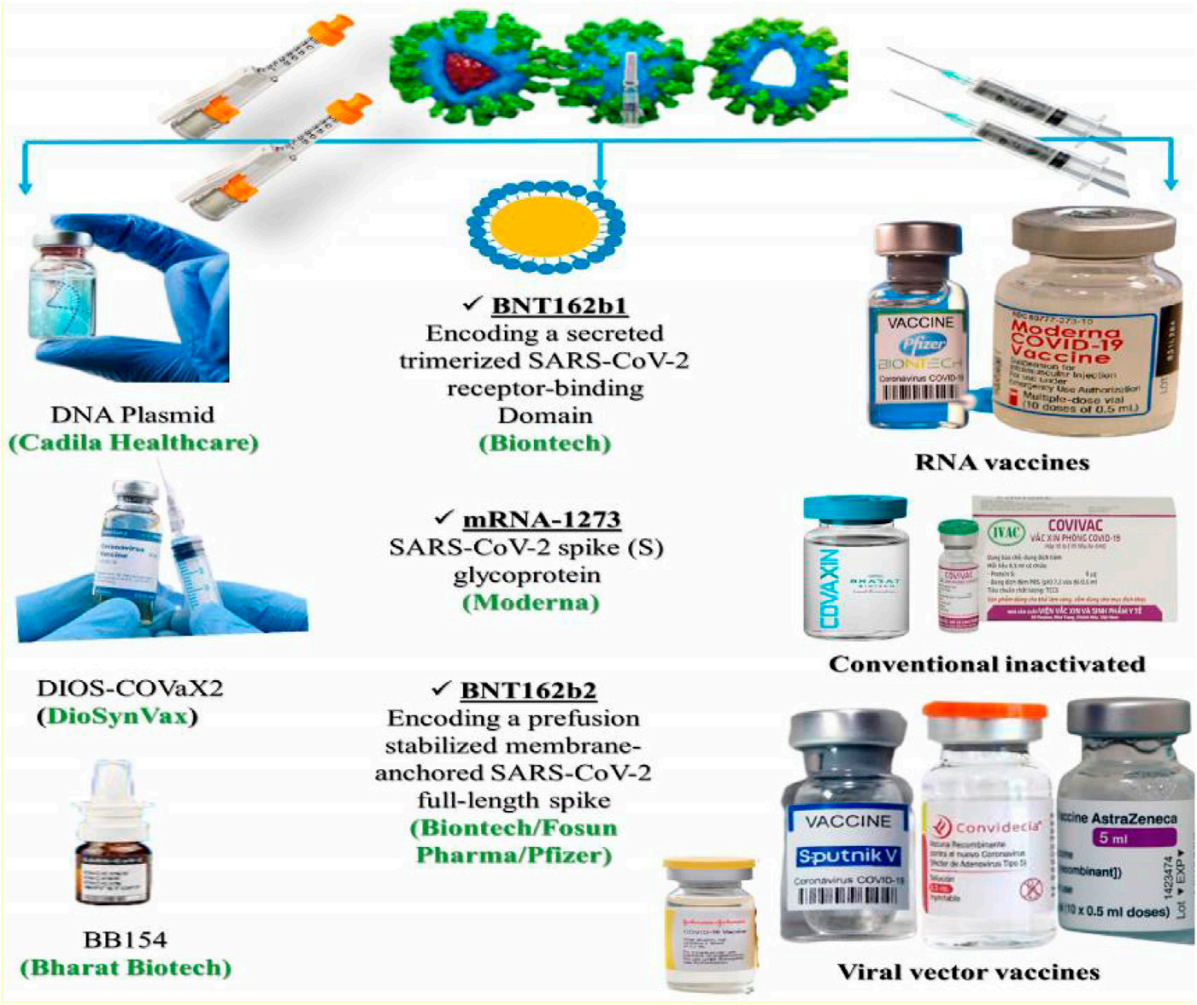
Portrayal of several coronavirus vaccinations, administered with or without needles, or using nanocarrier.

Since November 2019, a strongly transmissible coronavirus variant has been hitting the globe. COVID-19 infection has taken the lives of over 2 million people. The governments around the world have started mass vaccine campaigns using syringes and needles for COVID-19. Approximately 15 corona vaccines have been authorized globally (Figure 6), which include mRNA-based, viral vector-based, conventional inactivated, and protein-based vaccines. All these vaccines require the use of needles and are expensive. Production of these vaccines is also difficult as it necessitates technology that involves freezing and cold-chain transportation. Furthermore, it requires specialized personnel to administer vaccines through needles and results in long queues and squandered doses costing billions of dollars. Thus, a cost-effective technology that develops heat-stable vaccines, requires little administrator preparation, and overcomes the aforementioned limitations, is warranted. The mission for alternative approach against coronavirus is now underway and many pharmaceutical firms around the world have been involved in developing needle-less coronavirus vaccines using new delivery methods including implants, nasal spray, pills, patches and electrical pulse system.

The Cadila Healthcare Industry, based in Ahmadabad, India has developed a needle-less vaccine based on DNA plasmid using PharmaJet needle-free injection technology, which is administered through the skin. It does not penetrate the body deeply and is delivered using a special unit. It is administered without the use of needles and is painless. Furthermore, the firm claims that this vaccine would prove to be better than other vaccines as it is associated with lesser side effects. They are in the process of conducting phase 3 trials on over 20,000 people to determine whether the vaccine can be delivered in two doses instead of three via a needle-free injection, and if so, determine how many doses are required. The first and second doses will be delivered on days 1 and 28, and the third dose will be given on day 54 (Rajagopal, 2021). Two clinical phases have already been completed and both have shown the vaccine to be safe and successfully evoke good immunogenic responses.

DIOSynVax (Digitally designed, Immune Optimised Selected and Synthesized Vaccines), University of Cambridge also developed a PharmaJet needleless vaccine called DIOS-CoVax2 that can be delivered through patient’s skin using a single jet of air and does not cause pain (Diosynvax Ltd, 2020). The structure and genetic code of SARS-CoV-2 were analyzed using 3D computer modeling and a vaccine that is specific for evoking antibodies to this virus was developed. The 3D modeling showed that the coronavirus has a spherical form of spike proteins that attack cells of the body and the developed vaccine technique focuses on preventing this binding. The clinical trial for this vaccine that targets all coronaviruses would begin in the autumn of 2021. Enesi Pharma Ltd., an advanced biotechnology firm headquartered in Oxfordshire, England (executive David Hipkiss), has recently designed a needle-less heat-stable RNA-based vaccine against SARS-CoV-2 using Imperial’s Polyplex and ImplaVax technology. This technology allowed the advancement of fixed solid dose vaccines that are engineered to be administered into the dermal layer of the skin using a needle-free system. This prepared vaccine was shown to be stable at 104°F and might reduce cold chain requirements (Enesi Pharma, 2020).

COVI-VAC, a vaccine developed by Codagenix, also provided good protection against the current COVID-19 virus. It can be administered intranasally and does not require the use of a syringe or needle. It is a single-dose live-attenuated vaccine that can be given directly to the nasal mucosa where the coronavirus infection starts (CODAGENIX Vaccine Programs, 2021). COVI-VAC, unlike other vaccines, is intended to provide long-lasting cellular immunity to all coronavirus proteins including spike proteins. It is currently being tested in a phase 1 trial in the United Kingdom (Arthur, 2021). In addition, Bharat Biotech, India also prepared an intranasal route vaccine (BBVI54), which needs only a single dose and T cells, IgG, and IgA responses are all shown to be stimulated by this vaccine. They have already conducted preclinical trials on animals including hamsters, Rhesus macaques, and mice to stop the virus from infecting and spreading (Bricker et al., 2020). Coronavirus vaccines ingested orally, by nasal spray, or through skin are better choice for particular groups, such as pregnant women and others. In response to the pandemic, vaccine testing and manufacturing has accelerated and nanotechnology has been playing an important role in the fight against this virus. New researches on nanovaccine delivery are underway with the aim of monitoring and preventing the spread and reoccurrence of the virus. Various mRNA nano delivery-based vaccines modified into the lipid nanoparticles and administered intramuscularly are undergoing clinical trials. In younger and older adults, mRNA-based nanovaccine delivery against COVID-19 elicited high levels of binding and activated neutralizing antibodies. It is currently being tested in phase 1 and 2 trials, with no significant side effects identified so far.

## Patents on Oral Transmucosal and Intranasal Vaccines

Several patents have been granted on the development of oral transmucosal and intranasal vaccines which were both related to the process of development of vaccine as well as the final product. Several researchers used chitosan as an adjuvant to develop the vaccine for influenza virus intended for mucosal administration and in one such attempt, a pharmaceutical product was developed comprising of a dispensing device in order to deliver the vaccine intranasally. It was claimed that mucosal delivery of influenza vaccine was found to be significantly enhanced upon addition of chitosan as an adjuvant (Makin et al., 2003). The efficacy of chitosan as adjuvants greatly depends on the level of acetylation as the chitosan with low level of acetylation showed poor adjuvant properties and chitosan with acetylation level 80% or more had good adjuvant properties and are suitable to be used in the mucosal delivery of vaccine. Similarly, in another study, an intranasal vaccine delivery system was developed using capsular saccharide antigen and chitosan as a carrier (Giudice et al., 2006). This antigen was obtained from the serogroup C of *Neisseria meningitidis* or at least two of serogroups A, C, W135 and Y of *N. meningitidis*. The carrier protein should be conjugated to the capsular saccharides and conjugated oligosaccharides were more preferred. Another invention in 2008 revealed the development of an intranasal vaccine using chitosan as an adjuvant having a molecular weight ranging from 10 kD to 500 kD and antigen consisting of inactivated, disrupted, or live attenuated viruses (Illum and Chatfield, 2008). Chitosan was further utilized as adjuvant in another invention where the chitosan was cross-linked with an aldehyde or mannosylated chitosan and combined with the antigens (Hargis et al., 2014). Chitosan as an adjuvant, formulation of vaccines containing chitosan as adjuvant and methods of preparation of vaccines were described.

Another approach to encapsulate the antigens using nanocarriers was made when oral alginate microsphere vaccines with a size of less than 5 nm were successfully developed for oral mucosal administration (Jeong et al., 2001). The vaccine was prepared using diffusion-controlled interfacial gelation process, and the protein antigen was encapsulated in biodegradable alginate microspheres to achieve the desired result. Several other adjuvants have also been tried with various antigens for delivery through the mucosal route. These include gelatin, soy-lecithin, plant lectin, carbopol, etc. A recombinant vaccine intended to be administered through the mucosal route was developed using gelatin as adjuvant (Chang et al., 2009). Gelatin proved to be non-immunogenic and showed excellent proteolytic stability when used as an adjuvant in the development of mucosal vaccines. Similarly, a vaccine formulation made up of an immunogen and plan lectin as adjuvant was developed which was claimed to improve the immunological T cell response in a variety of mammalian species, including dogs, cats, mice, rats, rabbits, Guinea pigs, chimpanzees, baboons, and humans (O’Hagan and Lavelle, 2005). Another mucoadhesive vaccine consisting of α-lecithin and carbopol was developed that demonstrated excellent mucoadhesive and adsorptive characteristics of vaccine antigen onto the mucosal sites (Gerber, 2004).

Dry powder vaccine containing antigens along with microcrystalline cellulose having diameter in the range of 10–100 microns were prepared for the prevention of influenza virus infection and to be given through intranasal route (Nagata and Haruta, 2020). The specific antigens derived form VP1 polypeptide of the EV71 virus which causes the disease of hand, foot and mouth in humans were used to prepare the vaccine and were shown to exhibit excellent immune responses *in vivo* (Das et al., 2020). In another attempt, a vaccine particularly aimed for GIT was developed which get activated on the ileum and appendix. Antigens were contained in a capsule which was further encapsulated in another capsule with enteric coating. These capsules released their contents at pH equal to or more than 7 (Kabadu and Schentag, 2020). Fluorinated cyclic dinucleotide adjuvant was used as an adjuvant with non-replicating antigens for the preparation of oral vaccines which were used for the prevention of *Helicobacter pylori*, *Clostridium difficile* and *Listeria* infections (Yan et al., 2019). A fast dissolving dosage form was developed and used for the delivery of vaccine intended to be used against parasitic or protozoal illness (Tian and Mclaughlin, 2018). The developed dosage form contained an immune response-enhancing matrix-forming substance such as starch (7–75%) or gelatin (3–55%) or mannitol (7–65%). An edible vaccine was developed recently using transgenic soyabeans that was found to be effective for the prevention of cancer and infections due to viruses, bacteria, fungi and parasites (Piller and Bost, 2018).

In continuation with the development of nanocarrier-based mucosal vaccines, a nanoemulsion-based vaccine was invented which was both safe and effective against a wide variety of pathogens that have been exposed to the atmosphere (Baker et al., 2008). The same group of scientists successfully developed nanoemulsion-based vaccine against the hepatitis B virus that can be delivered intranasally. Nanoemulsions were used as adjuvants and were made from a combination of oil, water, surfactant, and cetylpyridinium chloride (Baker et al., 2016).

## Conclusions and Future Perspectives

Vaccination through transmucosal route is a needle-free and therefore medical waste-free strategy providing immunity in both mucosal and systemic compartments against a range of pathogenic bacteria and viruses. The mucosa is enriched with both lymphatic and vascular drainage and the pre-systemic elimination and first-pass metabolism can be avoided. A retentive device can easily be incorporated into the mucosal area and is well suited for patients of all age groups. Vaccine delivery through the mucosal route can further be improved using nanotechnology and by employing physical methods such as sonophoresis and iontophoresis. As the details of all mucosal sites are available, specific mucosal site for a particular situation can easily be selected. Needle-free vaccines are being explored extensively nowadays as they have the potential to increase the efficiencies, are adaptable to every operation, and can be altered in order to optimize their production. Various methods which can produce longer-lasting medications and therefore reducing the number of injections are also being investigated. However, safe and effective substances that enhance the buccal permeation and absorption are vital for the development of buccal drug delivery systems. Currently, commercially available formulations are mostly limited to tablets and films, while the buccal and sublingual routes offer great prospects and several formulation approaches are being investigated for this purpose. Previous studies reported that the nanoscale drug delivery devices hold significant potential in both clinical and industrial applications. Nanocarrier-based technologies are also being utilized to deliver antigens in an efficient, targeted, and controlled manner that is not achievable with traditional approaches. Polymeric nanoparticles, liposomes, virosomes, niosomes, and nanoemulsions are among the nano-sized delivery vehicles used in these technologies. Although, various types of nanocarrier-based formulations are being assessed for the mucosal administration of nonliving vaccines; most of them are still in the preclinical development phase and none has entered clinical trials. The vaccine delivery systems are gaining much popularity these days owing to their benefits in being patient-friendly, minimizing the need of booster doses and giving long-term treatment in modest doses.
